# Robot-assisted corpo-caudal pancreatectomy and splenectomy for pancreatic acinar cell carcinoma: a case report

**DOI:** 10.1093/jscr/rjaf112

**Published:** 2025-03-05

**Authors:** Muñoz Andrade Luis, Nevarez Francisco

**Affiliations:** National Oncology Institute SOLCA Guayaquil, Department of Digestive Surgery, Guayaquil 090505, Ecuador; National Oncology Institute SOLCA Guayaquil, Department of Digestive Surgery, Guayaquil 090505, Ecuador

**Keywords:** robotic pancreatectomy, acinar cell carcinoma, minimally invasive surgery, Da Vinci surgical system, pancreatic tumors

## Abstract

Acinar cell carcinoma of the pancreas (ACCP) is an exceptionally rare malignancy, accounting for <1% of all exocrine pancreatic tumors. This case report describes a 78-year-old male presenting with abdominal pain, weight loss, and anorexia, diagnosed with a tumor in the pancreatic body through imaging and biopsy. The patient underwent a robot-assisted corpo-caudal pancreatectomy and splenectomy using the Da Vinci system. This approach enabled precise resection with minimal blood loss and preservation of critical structures. Histopathology confirmed a well-differentiated acinar cell carcinoma with tumor-free margins. The postoperative course was uneventful, and follow-up imaging at three and six months demonstrated no recurrence or metastasis. This case underscores the advantages of robotic-assisted surgery, including enhanced precision, reduced complications, and optimized recovery, highlighting its role as a transformative tool for managing complex pancreatic tumors like ACCP.

## Introduction

Acinar cell carcinoma of the pancreas (ACCP) is a rare malignancy, accounting for <1% of all exocrine pancreatic tumors [1]. It arises from acinar cells responsible for digestive enzyme production and typically presents with nonspecific symptoms like abdominal pain, weight loss, and anorexia, leading to delayed diagnosis [2]. Compared to pancreatic ductal adenocarcinoma, ACCP exhibits less aggressive behavior and a more favorable prognosis with complete surgical resection [3].

Surgical resection remains the standard and potentially curative treatment for localized ACCP. However, the pancreas’s complex anatomy and proximity to vital structures pose significant challenges [4]. Advances in robotic-assisted surgery, particularly with the Da Vinci system, have revolutionized these complex procedures, offering enhanced precision, reduced blood loss, and faster recovery [5].

This report details a 78-year-old male with ACCP successfully treated with robot-assisted distal pancreatectomy and splenectomy, highlighting robotics’ potential in rare pancreatic tumor management.

## Case report

A 78-year-old male with no significant medical history presented with persistent abdominal pain, progressive weight loss of 5 kg over 4 months, and anorexia. No signs of jaundice, steatorrhea, or pancreatic insufficiency were identified. Laboratory tests revealed elevated amylase and lipase levels, with tumor markers, including CA 19–9 and CEA, within normal limits. Imaging confirmed a pancreatic body tumor.

### Preoperative evaluation

**Figure 1:**
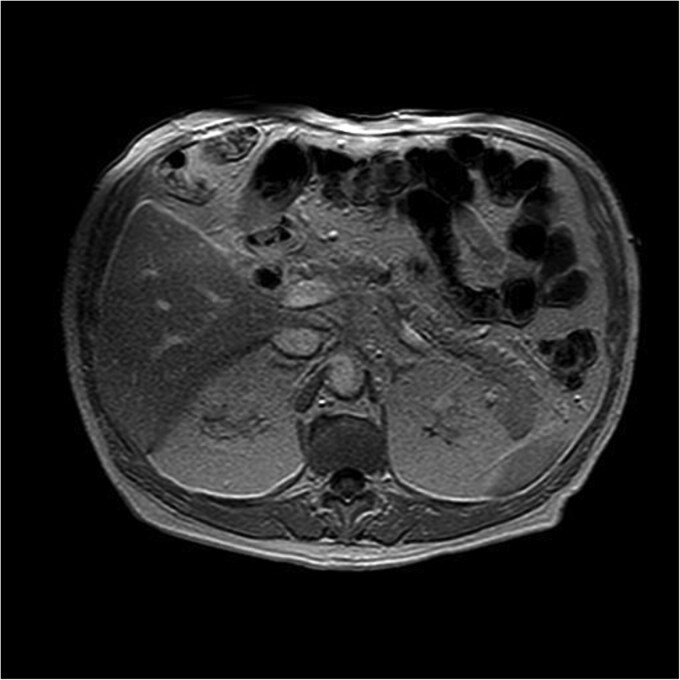
T1-weighted MRI illustrating a hypointense nodular lesion in the pancreatic tail, measuring 24 × 20 mm. The lesion demonstrates restricted diffusion and delayed contrast enhancement, consistent with a neoplastic process suggestive of malignancy.

Magnetic resonance imaging revealed a hypointense lesion (2.6 × 1.7 cm) in the pancreatic body on T1-weighted sequences, with restricted diffusion and delayed contrast enhancement, findings suggestive of malignancy. Endoscopic ultrasound with fine-needle aspiration confirmed the diagnosis of acinar cell carcinoma. There was no evidence of vascular invasion, lymph node involvement, or metastases (Fig. 1).

A multidisciplinary team deemed the patient an ideal candidate for surgical resection. A robot-assisted corpo-caudal pancreatectomy with splenectomy was planned to achieve complete tumor resection.

### Surgical procedure

**Figure 2:**
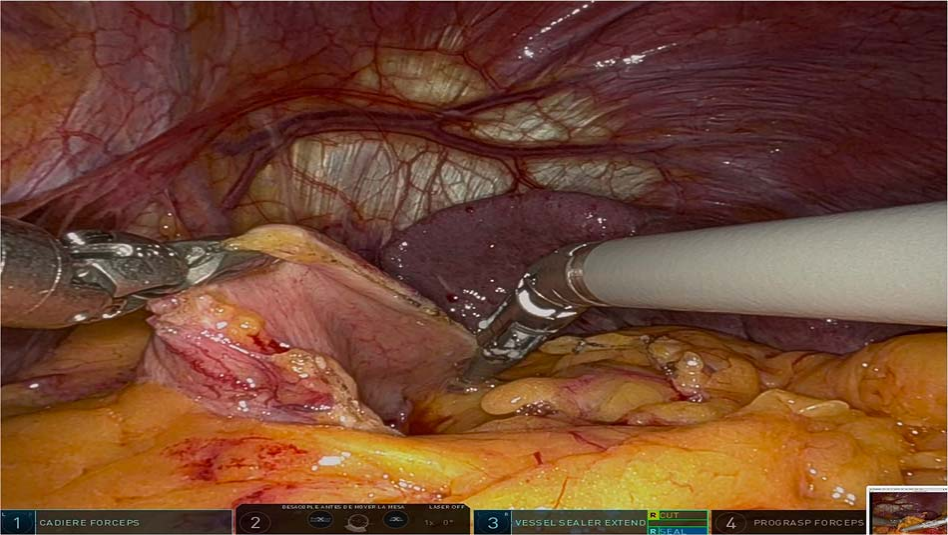
Intraoperative view showcasing the dissection and mobilization of the greater curvature of the stomach. This involved dividing the gastrocolic ligament to expose the retroperitoneal space, providing access to the distal pancreas while preserving surrounding structures to minimize surgical trauma.

**Figure 3:**
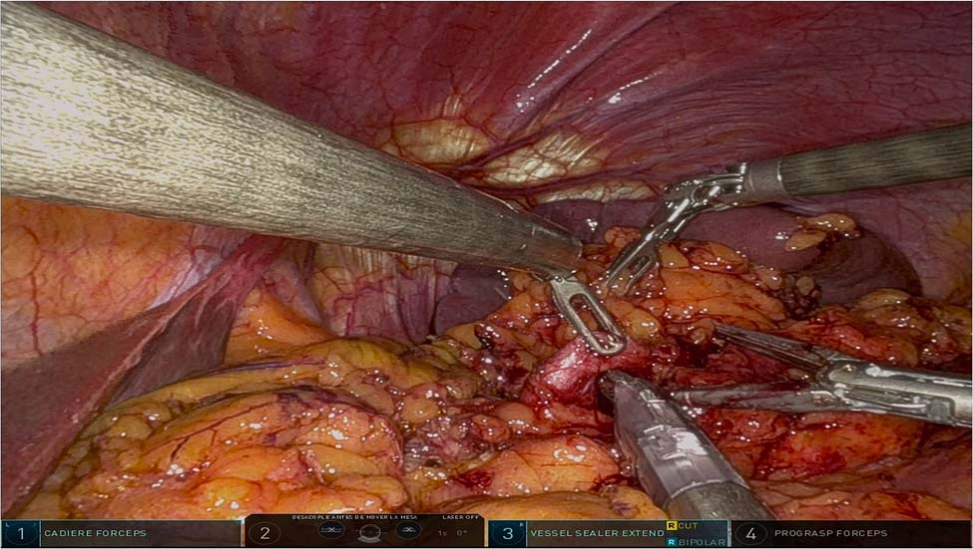
Intraoperative image highlighting the robotic ligation of the splenic artery. The Da Vinci system facilitated precise dissection and secure vascular control, minimizing blood loss and ensuring the preservation of adjacent structures, such as the splenic vein. This step was critical for the safe resection of the distal pancreas and spleen.

**Figure 4:**
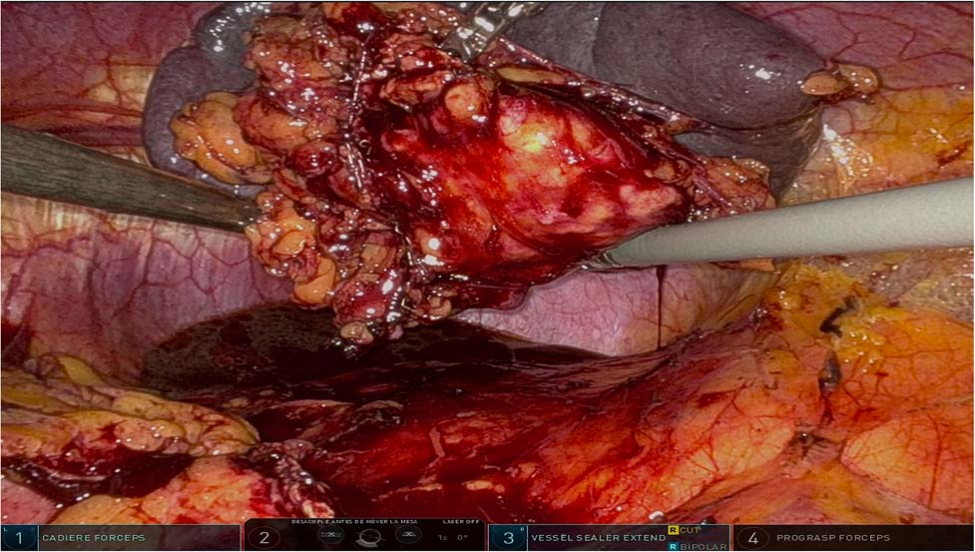
Complete surgical specimen, including the distal pancreas and spleen, shown after extraction via a mini-laparotomy. The specimen demonstrates clear resection margins, achieved through robotic precision, highlighting the effectiveness of the surgical approach.

The surgery was performed using the Da Vinci robotic system under general anesthesia. The patient was positioned supine with reverse Trendelenburg. Four robotic trocars and one assistant trocar were placed. The gastro-splenic ligament was dissected to expose the distal pancreas, and the pancreas was transected using a linear stapler, ensuring tumor-free margins (Fig. 2). The splenic artery and vein were individually ligated with robotic sutures, and the spleen was mobilized and removed along with the pancreas (Fig. 3). The specimen was extracted via mini-laparotomy and sent for histopathological analysis (Fig. 4).

The procedure lasted 180 minutes with 150 ml of blood loss. The robotic system’s precision minimized risks and preserved adjacent structures.

### Histopathological results

**Figure 5:**
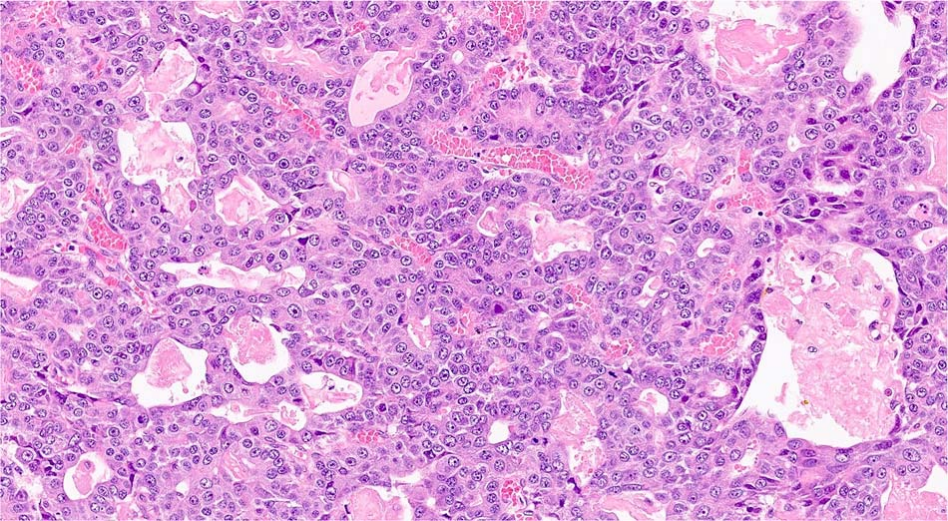
Histopathological slide showing well-differentiated acinar cell carcinoma with eosinophilic cytoplasm, monomorphic nuclei, and zymogen granules (hematoxilina-eosina staining, ×20 magnification).

The analysis confirmed a well-differentiated acinar cell carcinoma with no lymphovascular or perineural invasion (Fig. 5). Resection margins were tumor-free. Examination of 13 resected lymph nodes showed no metastatic involvement. The tumor was staged as pT2 N0 M0 (Stage IB).

### Postoperative course

Recovery was uneventful. The patient was mobilized on Day 1 and advanced to a normal diet without complications such as pancreatic fistula or infection. He was discharged on day four in good condition.

### Follow-up

**Figure 6:**
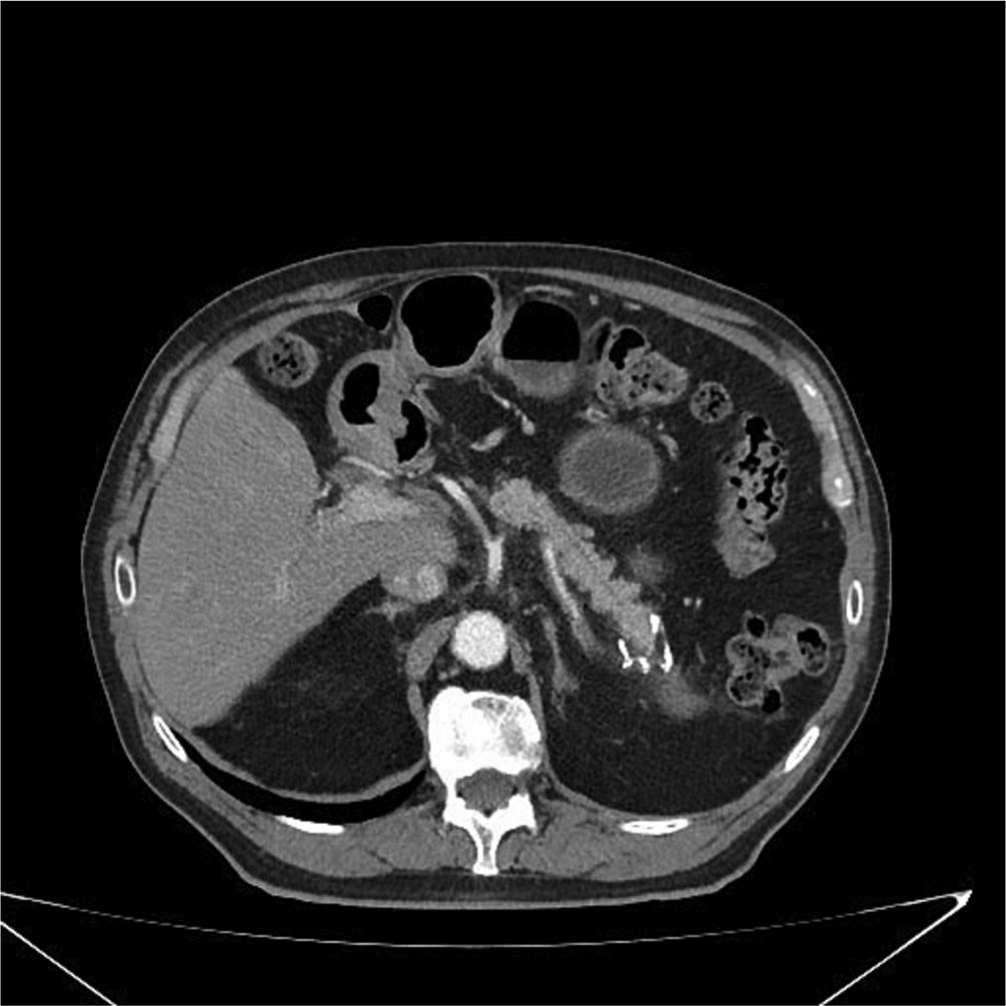
Postoperative CT scan showing surgical clips in the pancreatic tail region and a hypodense nodular lesion with lipomatous characteristics, measuring 2.1 cm, adjacent to the surgical site. No evidence of recurrence or complications is observed.

At 3 and 6 months postoperatively, CT showed no recurrence or metastases (Fig. 6). The patient remained asymptomatic with improved quality of life, including weight stabilization and resolution of abdominal pain.

### Clinical implications

This case highlights the effectiveness of robotic-assisted surgery in managing rare pancreatic tumors like ACCP. The Da Vinci system allowed for safe and precise tumor resection with minimal complications. This successful outcome supports the growing role of robotic surgery in complex pancreatic cases.

## Discussion

ACCP is an exceptionally rare malignancy, and its nonspecific clinical presentation often leads to delays in diagnosis. Unlike pancreatic ductal adenocarcinoma, ACCP tends to exhibit less aggressive behavior and offers a better prognosis following complete surgical resection [5]. Despite its rarity, achieving clear surgical margins remains a cornerstone for improved outcomes in localized cases [6].

Robotic-assisted surgery has transformed the management of complex pancreatic tumors, offering numerous advantages over traditional open and laparoscopic approaches. The Da Vinci robotic system provides enhanced three-dimensional visualization, superior dexterity, and greater precision, enabling surgeons to perform intricate dissections in anatomically challenging areas [7]. In ACCP cases, the ability to precisely transect and ligate the pancreas while preserving adjacent vital structures minimizes intraoperative risks and postoperative complications [8].

In this case, the use of the Da Vinci system facilitated a safe and effective robot-assisted corpo-caudal pancreatectomy and splenectomy. The procedure resulted in tumor-free surgical margins and an uneventful recovery. This aligns with existing literature highlighting the safety and efficacy of robotic-assisted approaches in pancreatic surgeries [9]. Studies have demonstrated that robotic techniques reduce blood loss, shorten hospital stays, and lower complication rates compared to open surgeries [10].

However, the adoption of robotic-assisted surgery is not without challenges. High costs, limited availability of robotic systems, and the need for specialized training represent significant barriers, particularly in developing countries [11]. Further multicenter studies and long-term follow-ups are needed to validate its cost-effectiveness and expand its application in rare malignancies like ACCP [12].

This case underscores the potential of robotic surgery in managing complex pancreatic tumors and supports its integration into modern surgical practice.

## References

[1] De Rooij T, van Hilst J, Bosscha K, et al. Minimally invasive surgery for pancreatic cancer. Best Pract Res Clin Gastroenterol 2016;30:941–51.

[2] Gagner M, Pomp A. Laparoscopic pylorus-preserving pancreatoduodenectomy. Surg Endosc 1994;8:408–10. 10.1007/BF00642443. 7915434

[3] Asbun HJ, Stauffer JA. Robotic-assisted pancreatic surgery: state of the art. J Gastrointest Surg 2012;16:2333–41.

[4] D'Angelo A, Caruso R, Persiani R, et al. The role of robotic surgery in pancreatic cancer. J Robot Surg 2013;7:11–8.

[5] Shrikhande SV, Barreto SG, Bodhankar YD, et al. Acinar cell carcinoma of the pancreas: a clinician's guide. J Gastrointest Oncol 2016;7:234–42.27034791

[6] Kim SC, Song KB, Hwang DW, et al. Comparison of laparoscopic and open distal pancreatectomy for left-sided ductal adenocarcinoma. Ann Surg Oncol 2011;18:3625–31.

[7] Moser AJ, Warren J, Fernandez-del CC. Robotic versus laparoscopic pancreatic surgery: which is better? Ann Surg 2017;266:1–3.27753648

[8] Zenati MS, Hamad A, Zeh HJ, et al. Robotic pancreatic surgery: outcomes and cost analysis. Minerva Chir 2018;73:89–98.

[9] Maggino L, Liu JB, Ecker BL, et al. Robotic versus laparoscopic distal pancreatectomy: a propensity score-matched analysis. J Gastrointest Surg 2019;23:1871–80.

[10] Waters JA, Canal DF, Wiebke EA, et al. Robotic distal pancreatectomy: cost-effective or cost-prohibitive? Ann Surg Oncol 2010;17:989–97. 10.1016/j.surg.2010.07.027 20797748

[11] Zureikat AH, Moser AJ, Boone BA. 250 robotic pancreatic resections: safety and feasibility. Ann Surg 2013;258:554–9. 10.1097/SLA.0b013e3182a4e87c. 24002300 PMC4619895

[12] Nassour I, Wang SC, Christie A, et al. Robotic pancreaticoduodenectomy: a critical appraisal. Surg Clin North Am 2020;100:231–46.

